# Matrix Hyaluronan Promotes Specific MicroRNA Upregulation Leading to Drug Resistance and Tumor Progression

**DOI:** 10.3390/ijms17040517

**Published:** 2016-04-07

**Authors:** Lilly Y. W. Bourguignon

**Affiliations:** San Francisco Veterans Affairs Medical Center, Department of Medicine, University of California at San Francisco & Endocrine Unit (111N2), 4150 Clement Street, San Francisco, CA 94121, USA; lilly.bourguignon@ucsf.edu; Tel.: +1-415-221-4810 (ext. 3321); Fax: +1-415-383-1638

**Keywords:** hyaluronan (HA), CD44, microRNA (miRNA), cancer stem cells (CSCs), signaling, chemoresistance, tumor progression

## Abstract

Solid tumor invasion, metastasis and therapeutic drug resistance are the common causes for serious morbidity and cancer recurrence in patients. A number of research studies have searched for malignancy-related biomarkers and drug targets that are closely linked to tumor cell properties. One of the candidates is matrix hyaluronan (HA), which is known as one of the major extracellular matrix (ECM) components. HA serves as a physiological ligand for surface CD44 molecule and also functions as a bio-regulator. The binding of HA to CD44 has been shown to stimulate concomitant activation of a number of oncogenic pathways and abnormal cellular processes in cancer cells and cancer stem cells (CSCs). MicroRNAs (miRNAs) belong to a class of small RNAs containing ~20–25 nucleotides and are known to promote aberrant cellular functions in cancer cells. In this article, I have focused on the role of HA interaction with CD44 and several important signaling molecules in the regulation of unique miRNAs (e.g., miR-21, miR-302 and miR-10b) and their downstream targets leading to multiple tumor cell-specific functions (e.g., tumor cell growth, drug resistance and metastasis) and cancer progression. This new knowledge could provide the groundwork necessary for establishing new tumor markers and developing important, novel drugs targeted against HA/CD44-associated tumor progression, which can be utilized in the therapeutic treatment of metastatic cancer patients.

## 1. Matrix Hyauronan (HA) and Tumor Progression

**Figure 1:**
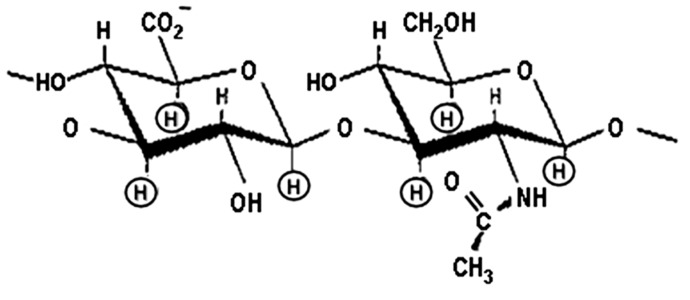
Illustration of the structure of hyaluronan (HA).

During the search for bio-regulators that associate with solid tumor cell functions, matrix hyaluronan was identified as one of the prime candidates [1,2]. Structural analyses indicate that hyaluronan (HA) consists of repeating disaccharide units, d-glucuronic acid and *N*-acetyl-d-glucosamine [3,4] and belongs to a class of nonsulfated, unbranched glycosaminoglycan (Figure 1). HA is highly enriched in solid tumors [5,6]. In breast cancer patients, a high level of HA accumulation occurs in malignant tumors as compared to the low level of HA accumulation in the benign or normal tissues [6]. In fact, the high level of HA accumulation is often used as a predictor of malignancy [6]. Elevated HA levels have also been detected in breast cancer patients’ serum [7].

Under physiological conditions, HA is produced by three HA synthases [8,9] and modified into smaller HA fragments by various hyaluronidases [10] (List 1). HA has been shown to be involved in modulating gene expression by epigenetic modifications [11]. Recent studies have shown that different sizes of HA (small-size HAs, <2 × 10^5^ Daltons) and their larger precursor molecules (*i.e.*, intact HA, >2–4 × 10^6^ Daltons) may be involved in distinct biological activities [12]. It has been suggested that the formation of biologically active HA fragments from the large HA (~7 × 10^5^–1 × 10^6^ Daltons) in the extracellular matrix (ECM) occurs during cell proliferation, migration and differentiation, as well as tissue repairs [12,13,14,15]. For example, large size-HA promotes transcriptional activation and differentiation, whereas small size-HA induces cell proliferation and migration [12,13,14,15]. HA has also been detected in stem cell niches [16,17]. The unique HA-enriched microenvironment appears to be involved in both self-renewal and differentiation of normal human stem cells [16,17]. Therefore, it is commonly believed that both abnormal HA synthesis and degradation contribute to aberrant cellular processes, such as tumor cell proliferation, migration and metastasis [5,6].

**List 1. Hyauronan (HA) synthases and hyaluronidases.**
HA Matrix-related Enzymes (Isozymes):
  HA Synthases: HAS-1, HAS-2 & HAS-3
  Hyaluronidases: Hyal-1, Hyal-2, Hyal-3, Hyal-4 & PH20

## 2. Hyaluronan Receptor, CD44 in Cancer Stem Cells (CSCs)

**Figure 2:**
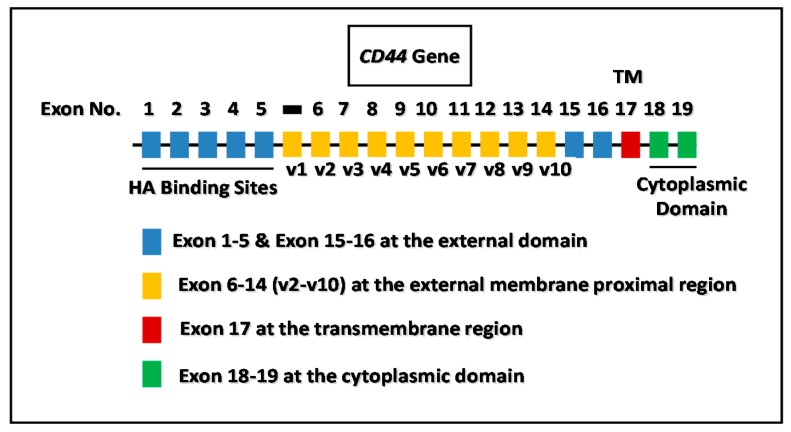
Illustration of *CD44* gene and alternative spliced variants (CD44v isoforms). The HA binding domain is located at the external (in particular, N-terminal) region of CD44 and the signaling regulators’ binding sites are located at the cytoplasmic domain of CD44.

CD44 has been characterized as a transmembrane glycoprotein and serves as a primary HA receptor in many cancer cells [18,19,20]. Nucleotide sequence analyses indicate that CD44 is present as multiple isoforms (via an alternative splicing mechanism), all of which are variants of the standard form of CD44, namely CD44s [21,22]. All CD44 isoforms [expressing exon 6–14, variant 1 (v1)–variant 10 (v10)] contain an HA-binding site at the N-terminal region of their extracellular domain (exon 1–5) and thereby serve as major cell surface receptors for HA [23,24,25,26]. The cytoplasmic domain of CD44 contains signaling regulators’ binding sites (exon 18–19) (Figure 2). HA binding to CD44 generates concomitant onset of multiple signaling pathways leading to tumor-specific behaviors and cancer progression [18,19,20]. A previous study indicated that downregulation of CD44 by treating cells with CD44 shRNA (a short-hairpin CD44-specific interference RNA) results in a reduction of liver tumor growth both *in vitro* and *in vivo* [27]. These results suggest that CD44 plays an important role in regulating cancer progression.

The CD44 expressed in some breast cancer cells displays unique properties to promote tumor cell-specific characteristics [28,29]. Further investigation indicates that these breast cancer tumors contain a subpopulation of highly tumorigenic cancer stem cells (CSCs) characterized by high CD44 expression and low (or no) CD24 expression (CD44^+^CD24^−/low^) [28,29]. Purified CD44^+^CD24^−/low^ breast tumor cells are capable of generating phenotypically distinct cells resulting in heterogeneous tumors in immunodeficient mice [28,29].

Recently, a high level of CD44v3 isoform together with aldehyde dehydrogenase-1 (ALDH1) (CD44v3^high^ALDH1^high^) have also been detected in the subpopulation of cancer stem cells (CSCs) of Human Head and Neck Squamous Cell Carcinoma (HNSCC) cell line [30,31]. These CSCs from HNSCC cells display the hallmark of stem cell properties including stem cell marker (e.g., Nanog, Oct4 and Sox2) expression, self-renewal/clonal formation and high tumorigenic in immunodeficient mice [30,31]. The fact that CD44 overexpressing tumor cells display the stem cell properties suggests that CD44 is an important cancer stem cell marker [28,29,30,31]. Previous studies indicated that overexpressed CD44 is frequently complexed with HA at the tumor attachment sites, and HA-CD44 interaction stimulates a variety of tumor cell-specific functions and cancer progression [18,19,20,30,31]. These findings suggest that the HA binding to CD44 in tumor cells is considered an essential requirement for tumor progression.

## 3. HA-CD44-Induced Oncogenic MicroRNAs (miRNAs) and Disease Progression

MicroRNAs (miRNAs) (consisting of ~21–25 nucleotides in length) are single-stranded RNAs which often participate in the modulation of gene expression at the posttranslational level [32]. In mammalian systems, both the binding between the “seed region” of the 5′-end of the miRNA and the 3′-untranslated region (3′-UTR) of the mRNAs contribute to the selection of miRNA-specific targets [33]. MicroRNAs (miRNAs) also play an important role in the regulation of gene expression during normal development. An estimated 30%–60% of the coding genes are modulated by miRNA-related silencing, which leads to abnormal miRNA expression in many diseases [34]. Further analyses indicated that approximately 50% of miRNAs are detected at malignancy-related genomic sites/fragile regions. These findings suggest that certain miRNAs may be closely associated with cancer progression [35,36]. A previous study showed that CD44 interaction with HER2 promotes CXCR4 overexpression by downregulating microRNA-139 (via epigenetic regulation) in gastric cancer cells [37]. Thus, CD44 appears to be tightly linked to miRNA regulation.

### 3.1. HA-CD44 Interaction Promotes miRNA-21 Expression and Chemoresistance

MicroRNA-21 (miR-21) is one of the most studied miRNAs in recent cancer research. The miRNA array data indicate that miR-21 is frequently overexpressed in tumors compared with normal tissues [35,36]. The physiological importance of miR-21 has also been demonstrated in a number of studies following the identification of its specific targets [38]. For example, miR-21 binds to a conserved site within the 3′-untranslated region of mRNA of the program cell death 4, PDCD4 (a tumor suppressor protein) leading to PDCD4 downregulation and tumor cell invasion and metastasis [38,39]. Overexpression of miR-21 also participates in the modulation of tumor cell-specific properties including cell proliferation, invasion, metastasis and chemotherapeutic drug resistance in a variety of cancer cells [39,40,41]. HA binding to CD44 stimulates miR-21 expression has been reported in several cancer cells including breast cancer cells [39] and head and neck cancer cells [42]. This event contributes to not only an upregulation of survival proteins, such as cIAP-1/cIAP-2/XIAP (IAPs, inhibitors of apoptosis proteins) [42], but also an overexpression of the multidrug resistant proteins required for cell survival and chemotherapy resistance in tumor cells [39,40,41,42]. Currently, miR-21 is considered to be an oncogene.

The question of how HA/CD44-mediated miR-21 is regulated in cancer cells is described as follows:

#### 3.1.1. Nanog, PKCϵ and Stat-3 Signaling Pathway in Regulating HA/CD44-Mediated miR-21:

Nanog (a stem cell marker) is one of the transcription factors involved in the self-renewal and pluripotency maintenance of embryonic stem (ES) cells and mammalian embryo development [43]. Signaling interaction between Nanog and various stem cell regulators (e.g., Rex1, Sox2 and Oct3/4) activates the expression of several target genes needed for pluripotency of ES cells [43]. These findings suggest that Nanog plays an essential role in in regulating many cellular activities. The expression of Nanog is also detected in many tumor cell types [44,45]. Recently, it has been shown that the binding of HA to breast tumor cells promotes CD44-Nanog complex formation followed by the activation of miRNA signaling [39]. These findings strongly suggest that HA-CD44 interaction is tightly associated with Nanog-regulated miRNA function.

Further analyses indicate that HA binding to CD44 stimulates PKCε activation and Nanog phosphorylation, which in turn, activates Nanog signaling-regulated miR-21 production in breast cancer cells. These events contribute to the reduction of PDCD4 (the tumor suppressor protein), overexpression of IAP/MDR1 (P-gp), cell survival, and chemotherapeutic drug resistance in breast tumor cells. Inhibition of PKCϵ-Nanog signaling by downregulating PKCε with PKCϵ siRNA or silencing miR-21 with anti-miR-21 inhibitor results in PDCD4 up-regulation and causes IAP/MDR1 (P-gp) reduction, as well as apoptosis/cell death and chemosensitivity in breast tumor cells [39]. These findings provide important new insights into understanding the roles that HA-CD44-mediated PKCϵ activation and Nanog-regulated miR-21 play in regulating anti-apoptosis and chemotherapy resistance in breast tumor cells.

**Figure 3:**
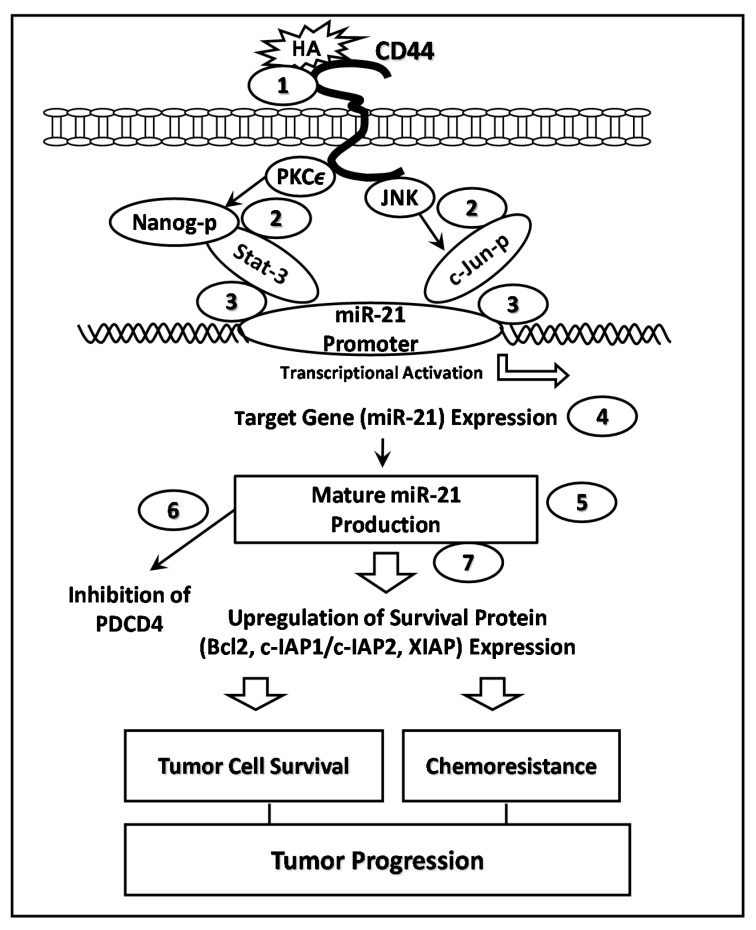
PKCϵ and JNK (c-Jun-NH2-Kinase) Signaling Pathways in Regulating HA/CD44-mediated miR-21 expression leading to chemoresistance: In the PKCϵ signaling pathway, HA binding to CD44 (step 1) stimulates PKCϵ activity (step 2), which, in turn, causes Nanog phosphorylation and Nanog-Stat-3 complex association with miR-21 promoter (step 3) leading to transcriptional activation (step 4) and the expression of miR-21 (step 5). Upregulation of miR-21 then decreases the expression of the tumor suppressor protein, PDCD4 (step 6) and increases the expression of IAP (survivin and XIAP)/MDR1 (P-gp), tumor cell anti-apoptosis/survival, and chemoresistance (step 7). All these events are required for cancer progression. In the JNK/c-Jun pathway, the binding of HA to CD44 (step 1) stimulates JNK and c-Jun phosphorylation (step 2). Subsequently, phosphorylated c-Jun binds to the miR-21 promoter (step 3), resulting in transcriptional activation (step 4) and mature miR-21 production (step 5). Upregulation of miR-21 then reduces the tumor suppressor protein, PDCD4 *(*step 6*)* and increases the expression of the survival proteins (e.g., Bcl2, c-IAP-1, c-IAP-2 and XIAP) (step 7), anti-apoptosis/survival and chemo resistance. All these events are needed for cancer progression.

Hyaluronan (HA) also induces CD44 binding to Nanog and Stat-3 (a transcription factor) in head and neck cancers (HNSCC, HSC-3 cells line). Specifically, HA-CD44 binding to HNSCC cells promotes Nanog-Stat-3 (also tyrosine phosphorylated Stat-3) complex formation and transcriptional activation. The results of a ChIP assay indicate that HA/CD44-induced Nanog-Stat-3 complexes become recruited into the promoter enhancer region of miR-21 in HNSCC cells [42]. This process results in miR-21 gene expression/production, a reduction of PDCD4 (a tumor suppressor protein), and an upregulation of survival proteins as well as an enhancement of cisplatin-resistance in HNSCC cells [42]. Downregulation of HNSCC cells with Nanog/Stat-3 and/or miR-21 by treating cells with Nanog/Stat-3-specific siRNAs or anti-miR-21 inhibitor effectively blocks HA-mediated Nanog-Stat-3 signaling events, abrogates miR-21 expression and increases PDCD4 expression. These events then lead to survival protein expression and enhancement of chemosensitivity to cisplatin in HA/CD44-treated HNSCC cells. Together, these findings strongly suggest that the HA-induced CD44 interaction with Nanog and Stat-3 plays a pivotal role in miR-21 gene expression/production required for tumor suppressor protein reduction, survival protein overexpression and cisplatin resistance in HNSCC cells. Consequently, miR-21 expression regulated by Nanog/Stat-3 signaling pathway could be useful for the future drug target designs to treat HA/CD44-activated HNSCC (Figure 3). 

#### 3.1.2. c-Jun Signaling Pathway in the Regulation of miR-21:

HA-mediated CD44 signaling plays important roles in the regulation of solid tumor malignancies such as breast cancer [18]. Activation of JNK and c-Jun signaling pathway has been shown to be involved in the onset of breast cancer [46,47]. Phosphorylation of JNK and c-Jun is required for the activation of a variety of genes [48,49]. Specifically, c-Jun phosphorylation at Ser-63 [pS63] residues by JNK is known to occur within the transcriptional activation region of c-Jun [36]. A previous study indicated that miR-21 gene expression/production can also be regulated by HA/CD44-mediated phosphorylation of JNK/c-Jun and JNK-c-Jun pathway in MDA-MB-468 cell line (a triple negative breast cancer cell line). The results of ChIP assays indicate that the binding of HA to CD44 stimulates the recruitment of c-Jun/phosphorylated c-Jun into the promoter region of miR-21. These events then lead to the expression of miR-21 and upregulation of survival proteins (IAPs and Bcl-2), as well as Doxorubicin chemoresistance in MDA-MB-468 cells. Inhibition of JNK/c-Jun signaling or silencing miR-21 production causes IAPs/Bcl-2 reduction and chemosensitivity to Doxorubicin [50]. Thus, these findings strongly support the hypothesis that a functional signaling axis consisting of HA/CD44-regulated JNK/c-Jun and miR-21 is involved in the regulation of tumor cell survival and Doxorubicin chemoresistance in breast cancer cells (Figure 3). Recently, our preliminary data indicate both Stat-3/Nanog and JNK/c-Jun pathways can be simultaneously activated in the upregulation of miR-21 expression during HA/CD44-mediated signaling in both breast cancer cells and head and neck cancer cells.

### 3.2. HA-CD44 Interaction Promotes miRNA-302 Expression and Chemoresistance in Cancer Stem Cells (CSCs):

The biological functions of certain miRNAs are often involved in the regulation of self-renewal and/or differentiation [51,52,53]. Previous genetic studies using mouse models revealed that three transcription factors, namely Nanog, Oct4 and Sox2 have distinct roles but often use similar signaling pathways to maintain stemness functions during development [51,52,53]. Some studies also indicated that Nanog, Oct4 and Sox2 co-occupy the promoter of miR-302, which has been shown to target genes required for development and oncogenesis [54,55,56]. For example, Nanog, Oct4 and Sox2, form a positive autoregulatory loop to maintenance cells at an undifferentiated state. At the post-translational level, the miR-302 family also plays a key role in the regulation of cell proliferation and cell differentiation [54,55,56].

Recently, a unique subset of highly tumorigenic HNSCC cells that express high levels of CD44v3 and aldehyde dehydrogenase-1 (ALDH1) (designated as CD44v3^high^ALDH1^high^ cells) has been identified and characterized [30]. These CD44v3^high^ALDH1^high^ cells appear to exhibit CSC-like phenotypes [29]. Further analyses revealed a new HA/CD44-mediated signaling pathway that regulates stem cell marker (e.g., Nanog, Oct4 and Sox2)-associated miR-302 production in CD44v3^high^ALDH1^high^ CSCs. Specifically, the HA-CD44v3 interaction activates Nanog, Oct4 and Sox2 complex formation in CD44v3^high^ALDH1^high^ cells [30]. Furthermore, the miR-302 cluster appears to be controlled by a promoter containing Nanog-Oct4-Sox2-binding sites in these cells, whereas ChIP assays demonstrate that stimulation of miR-302a and miR-302b production by HA is Nanog/Oct4 and Sox2 complex-dependent in CD44v3^high^ALDH1^high^ cells [30]. Importantly, overexpression of miR-302a and miR-302b occur in both mouse tumors induced by CD44v3^high^ALDH1^high^ cells and human HNSCC patient samples [29]. These findings clearly established a close relationship between miR-302 clusters (e.g., miR-302a and miR-302b) and HNSCC development.

**Figure 4:**
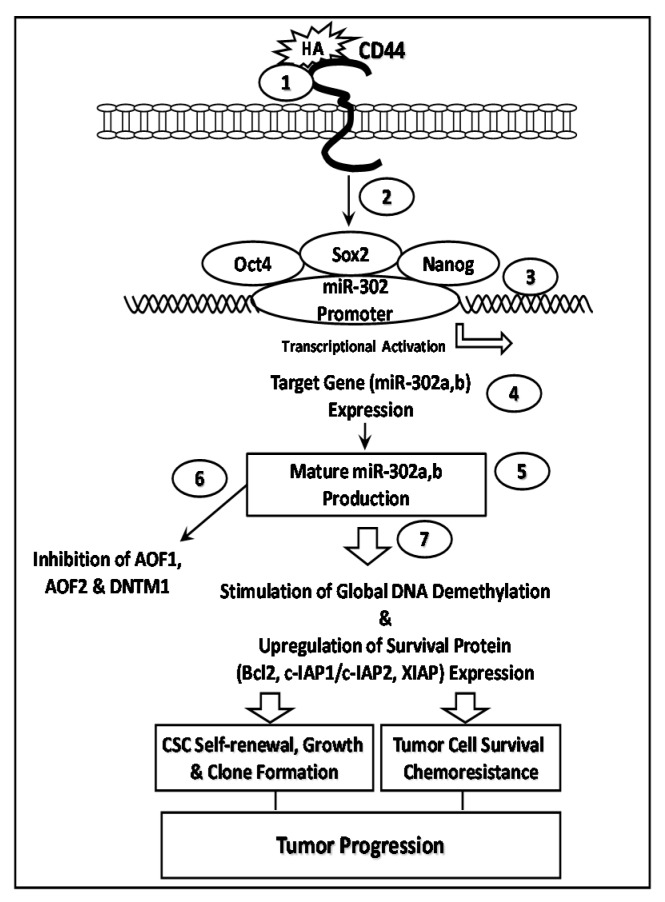
HA-CD44 interaction promotes miRNA-302 expression and chemoresistance: In cancer stem cell (CSC) signaling, the binding of HA to CD44 (step 1) stimulates Nanog, Oct4, and Sox2 complex formation with CD44v3 (step 2)*.* This Nanog-Oct4-Sox2 signaling complex then interacts with the promoter region (containing Nanog, Oct4 and Sox2-binding sites) of the miR-302 cluster (step 3) resulting in miR-302 cluster gene expression (step 4) and mature miR-302a and miR-302b production (step 5)*.* The resultant miR-302 then downregulates the lysine-specific histone demethylases (namely AOF1 and AOF2) and DNA (cytosine-5)-methyltransferase 1 (DNMT1) (step 6) and induces global DNA demethylation (step 7) leading to IAP (cIAP-1, cIAP-2, and XIAP) expression, self-renewal, clonal formation, anti-apoptosis/survival, and chemoresistance in CSC-like CD44v3^high^ALDH1^high^ cells. All these events participate in cancer progression.

Furthermore, HA-CD44-activated miR-302 has been shown to regulate several cellular events including (i) the reduction of AOF1/AOF2 and DNMT1 (known as epigenetic regulators) resulting in global DNA demethylation; and (ii) overexpression of several survival proteins leading to self-renewal, clonal formation and cisplatin resistance. Inhibition of miR-302 expression/function not only results in upregulation of AOF1/AOF2/DNMT1 and reduction of global DNA demethylation, but also causes a decrease in the expression of cIAP-1/cIAP-2/XIAP and inhibition of CSC functions (e.g., self-renewal, clonal formation and cisplatin resistance) [30]. These findings suggest that cancer stem cell-specific properties are regulated by a novel HA/CD44-mediated Nanog/Oct4/Sox2 signaling and miR-302 in head and neck cancer (Figure 4).

### 3.3. HA-CD44 Interaction Upregulates miR-10b Expression Leading to Tumor Cell Migration/Invasion and Chemoresistance:

MicroRNA-10b was previously found to be upregulated together with overexpression of RhoC and urokinase-type plasminogen activator receptor during malignant glioma invasion and migration [57]. One of the cellular targets for miR-10b, KLF4 (Kruppel-like factor 4) has been identified in human esophageal cancer cell lines [58]. Moreover, miR-10b has been reported to promote invasion and metastasis in breast cancer cells [59]. An earlier report showed that inhibition of miR-10b expression in breast tumor cells treated with an anti-miR-10 inhibitor significantly decreases miR-10b expression and reduces HOXD10 (a miR-10b target) expression, leading to a significant reduction in mammary tumor progression both *in vitro* and *in vivo* [60]. Thus, miR-10b appears to be closely involved in the regulation of tumor metastasis.

Twist is a major regulator of embryonic morphogenesis and acts as a basic transcription factor protein [61]. It often binds to the promoter’s E-boxes (5′-CANNTG-3′ conserved sequences) via dimerization with other basic helix-loop-helix proteins for gene activation [62]. A recent study indicates that Twist expression is induced by c-Src kinase activation [63]. The elevated Twist expression plays an important role in angiogenesis and tumor progression [64]. Twist overexpression promotes a cancer stem cell (CSC) phenotype and has also been shown to induce miR-10b, which inhibits the mRNA of HOXD10 and results in the increase of RhoC in CD44-positive breast tumor cells [59]. Thus, Twist appears to have an important role in cancer progression. Understanding the mechanisms involved in c-Src-regulated Twist signaling and the subsequent expression of miR-10b is critically important for elucidating the mechanisms involved in HA/CD44-associated breast cancer metastasis.

**Figure 5:**
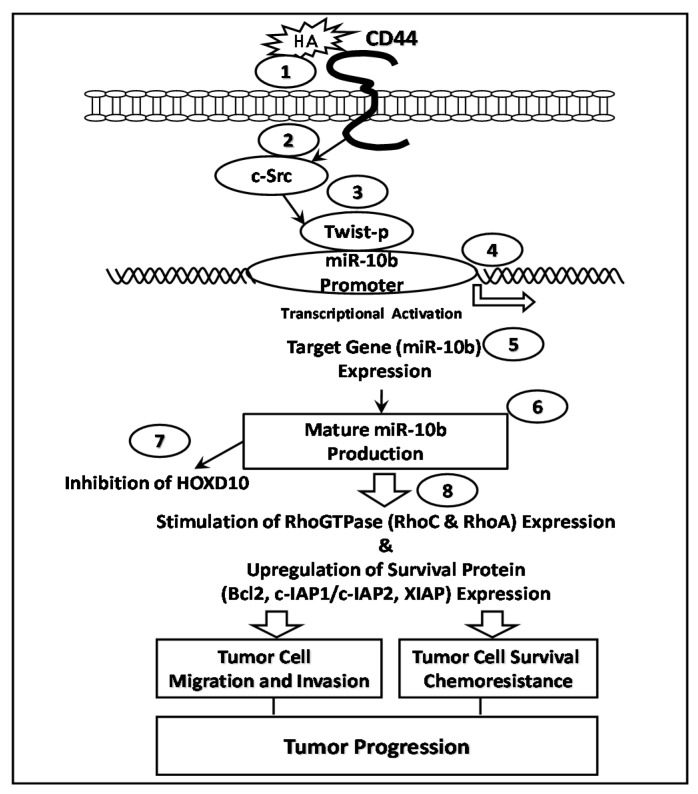
HA-CD44 interaction promotes c-Src and Twist activation and miR-10b expression during tumor cell migration/invasion and chemoresistance: In the c-Src-Twist signaling pathway, HA binding to CD44 (step 1) activates c-Src kinase (step 2), which, in turn, induces Twist phosphorylation (step 3)*.* Phosphorylated Twist then binds to the E-box elements of the mR-10b promoter (step 4)*,* causing transcriptional activation (step 5) and miR-10b expression (step 6). The upregulation of miR-10b then reduces the expression of HOXD10 (the tumor suppressor protein) (step 7) and enhances RhoA/RhoC upregulation and ROK activation, as well as cytoskeleton reorganization) leading to tumor metastasis and cancer progression.

A recent report indicated that a new HA/CD44-mediated signaling mechanism is involved in the regulation of Twist-associated miR-10b production and breast tumor cell invasion [65]. These results showed that HA/CD44-activated c-Src stimulates Twist phosphorylation, which, in turn, stimulates Twist-regulated miR-10b production. These events lead to the reduction of the tumor suppressor protein, HOXD10, RhoA/RhoC overexpression, ROK activation, and breast tumor cell invasion. Downregulation of c-Src/Twist or inhibition of miR-10b production results in HOXD10 upregulation and causes a decrease in the expression of RhoA/RhoC and a reduction of ROK-mediated breast tumor cell invasion. These findings strongly support the contention that a functional signaling axis consisting of c-Src, Twist, and miR-10b regulates RhoGTPase-ROK function and cytoskeleton-associated breast cancer metastasis [65] (Figure 5). Most recently, a study showed that different HA-size fragments (e.g., 5, 20, 200, or 700 kDa-HA-sizes) play a role in regulating CSCs isolated from head and neck cancer (HNSCC) cells. Specifically, 200 kDa-HA (but not other sizes of HA) preferentially induces certain stem cell marker expression resulting in self-renewal and clonal formation of HNSCC’s CSCs [31]. Further analyses indicate that 200 kDa-HA selectively stimulates the expression of a panel of microRNAs (most noticeably miR-10b) in these HNSCC’s CSCs. Survival protein, cIAP-1 expression was also stimulated by 200 kDa-HA in these HNSCC’s CSCs leading to cisplatin resistance. Furthermore, our results indicate that the anti-miR-10 inhibitor not only decreases survival protein expression, but also increases chemosensitivity of the 200 kDa-HA-treated HNSCC’s CSCs. Thus, 200 kDa-HA appears to have a key role in the regulation of miR-10 needed for survival protein expression and cisplatin resistance in HNSCC’s CSCs [65]. Together, these findings strongly suggest that oncogenic signaling-induced by certain sizes of HA (e.g., 200 kDa-HA) may be instrumental in the formation of CSC functions leading to tumor cell survival and chemoresistance in head and neck cancer progression (Figure 5).

## 4. Conclusions

Our current knowledge indicates that HA promotes CD44 interaction with a number of intracellular regulators in a variety of cancer cells. In particular, the “cross-talk” among various signaling regulator-associated pathways causes a concomitant onset of multiple cellular functions. In particular, the coordinated activation of miRNAs (e.g., miR-21, miR-302 and miR-10b) and various signaling regulators by HA-CD44 interaction as illustrated in three models (Figure 3, Figure 4 and Figure 5) could contribute to a variety of tumor cell-specific behaviors, tumor metastasis, progression and chemoresistance.

These HA/CD44-activated signaling regulators (PKCε, JNK, c-Src, Nanog, Stat-3, c-Jun, Oct4, Sox2 and Twist), together with various miRNAs could be used as structure/function-related tumor markers for human cancer detection and prognosis. In addition, downregulation of CD44 by lentiviral vector-based CD44 shRNAs and a miRZipsTM lentiviral-based miRNA inhibitor can specifically inhibit CD44 expression, as well as CD44-signaling-associated miRNAs (e.g., miR-21, miR-302 and miR-10b) expression and chemoresistance during solid tumor (e.g., breast, ovarian and head and neck cancer progression. These proposed signaling perturbation approaches could cause synergistic apoptotic responses and demonstrate that chemotherapy combined with the suppression of CD44, various signaling activators and miRNA inhibitors may be more effective than chemotherapy alone. We believe that these proposed models (Figure 3, Figure 4 and Figure 5) will provide the groundwork necessary for developing important, new drugs targeted against HA/CD44-associated tumor progression, which can be utilized in the therapeutic treatment of metastatic cancer patients.
